# Metabolic Alterations Caused by Defective Cardiolipin Remodeling in Inherited Cardiomyopathies

**DOI:** 10.3390/life10110277

**Published:** 2020-11-11

**Authors:** Christina Wasmus, Jan Dudek

**Affiliations:** Comprehensive Heart Failure Center (CHFC), University Clinic Würzburg, 97078 Würzburg, Germany; Wasmus_C@ukw.de

**Keywords:** cardiolipin, mitochondria, Barth syndrome, Sengers syndrome, respiratory chain, Dilated Cardiomyopathy with Ataxia, cardiomyopathy

## Abstract

The heart is the most energy-consuming organ in the human body. In heart failure, the homeostasis of energy supply and demand is endangered by an increase in cardiomyocyte workload, or by an insufficiency in energy-providing processes. Energy metabolism is directly associated with mitochondrial redox homeostasis. The production of toxic reactive oxygen species (ROS) may overwhelm mitochondrial and cellular ROS defense mechanisms in case of heart failure. Mitochondria are essential cell organelles and provide 95% of the required energy in the heart. Metabolic remodeling, changes in mitochondrial structure or function, and alterations in mitochondrial calcium signaling diminish mitochondrial energy provision in many forms of cardiomyopathy. The mitochondrial respiratory chain creates a proton gradient across the inner mitochondrial membrane, which couples respiration with oxidative phosphorylation and the preservation of energy in the chemical bonds of ATP. Akin to other mitochondrial enzymes, the respiratory chain is integrated into the inner mitochondrial membrane. The tight association with the mitochondrial phospholipid cardiolipin (CL) ensures its structural integrity and coordinates enzymatic activity. This review focuses on how changes in mitochondrial CL may be associated with heart failure. Dysfunctional CL has been found in diabetic cardiomyopathy, ischemia reperfusion injury and the aging heart. Barth syndrome (BTHS) is caused by an inherited defect in the biosynthesis of cardiolipin. Moreover, a dysfunctional CL pool causes other types of rare inherited cardiomyopathies, such as Sengers syndrome and Dilated Cardiomyopathy with Ataxia (DCMA). Here we review the impact of cardiolipin deficiency on mitochondrial functions in cellular and animal models. We describe the molecular mechanisms concerning mitochondrial dysfunction as an incitement of cardiomyopathy and discuss potential therapeutic strategies.

## 1. Introduction

The adult heart shows the highest metabolic activity of all organs in the human body by consuming 6 kg of ATP every day. It converts chemical energy stored in fatty acids, lactate and glucose into the mechanical energy to pump blood through the body. The heart contains the highest amount of mitochondria of any tissue [1], comprising about 35% of the cardiac myocyte cell volume [2,3]. A total of 95% of the energy demand of the heart is covered by oxidative phosphorylation in the mitochondria. Due to their central role in energy metabolism, a defect in mitochondria endangers the tight homeostasis of energy supply and demand in the heart. An imbalance between available energy and energy demand has been observed in almost all etiologies of heart failure [4]. Mitochondrial dysfunction can have deleterious consequences for the heart physiology and affects many forms of heart disease [5,6]. Dysfunctional mitochondria in skeletal muscle also impact heart failure and are associated with exercise intolerance [7].

Mitochondria are double membrane-surrounded organelles. The inner mitochondrial membrane forms cristae structures, which harbor the respiratory chain and form independent units of oxidative phosphorylation [8]. The respiratory chain consists of four complexes (complex I–IV), that are involved in the electron transport from NADH or FADH_2_ onto molecular oxygen. Electron transport is coupled with proton export across the inner membrane. The corresponding membrane potential is the driving force for the fifth complex, F_1_F_o_-ATP synthase, to produce ATP. The ADP/ATP carrier (ANT) ensures the exchange of ATP and ADP across the inner membrane.

The reducing equivalents, NADH or FADH_2_, are yielded in the mitochondrial Krebs cycle and NADH is additionally yielded in glycolysis. A high energy demand results in elevated levels of ADP, which accelerates ATP production at the F_1_F_o_-ATP synthase and thus increases the activity of the respiratory chain. To avoid draining of reducing equivalents under conditions of high energy demand, production is increased by a compensatory upregulation of the Krebs cycle. Ca^2+^ plays a key role in coupling energy demanding processes of the myofilaments with mitochondrial metabolism. During excitation, a contraction coupling release of cytosolic Ca^2+^ from the sarcoplasmic reticulum stimulates energy conversion in myofilaments. Additionally, Ca^2+^ is transported from the cytosol into mitochondria by the mitochondrial calcium uniporter (MCU). Mitochondrial Ca^2+^ potently activates several mitochondrial dehydrogenases of the Krebs cycle. This direct coupling of cytosolic and mitochondrial signals allows an immediate activation of Krebs cycle flux under conditions of increased workload. Besides their function in energy conversion, mitochondria participate in multiple metabolic pathways, such as the urea cycle, the metabolism of amino acids and lipids, and the biogenesis of heme and iron sulfur clusters. Mitochondria morphology is highly dynamic and maintained by fission and fusion processes. Mitochondrial dynamics are instrumental for many signaling pathways, such as programmed cell death, calcium signaling or innate immune responses. Impaired mitochondrial dynamics promote mitophagy. This mitochondria-specific form of autophagy maintains mitochondrial function which is particularly important for cardiac homeostasis [9].

Most of the key metabolic enzymes of the mitochondria are embedded in the inner membrane. The phospholipid Cardiolipin (CL) is the characteristic lipid of the inner membrane, playing a pivotal role in most mitochondrial metabolic activities. Here, we will discuss how CL is involved in many essential mitochondrial functions including morphology, metabolism and respiration. Defects in the biosynthesis and remodeling of CL have a strong impact on mitochondrial function and particularly affect tissues with a high energetic contribution of mitochondria, such as the heart and neuronal tissue. Diseases with a direct link to CL biosynthesis and remodeling comprise Sengers disease (OMIM 212350), Barth syndrome (OMIM 302060) and Dilated Cardiomyopathy with Ataxia (DCMA, OMIM 610198). Changes in CL levels are also involved in other cardiac modifications including ischemia/reperfusion injury, diabetic cardiomyopathy and the aging heart.

## 2. CL Biosynthesis

**Figure 1:**
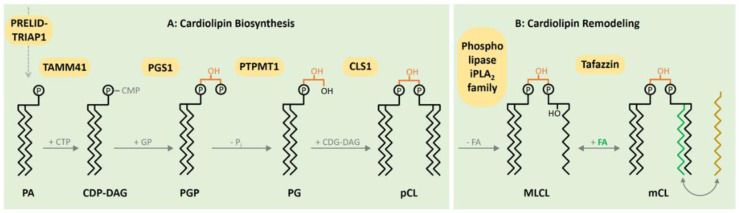
Biosynthesis and remodeling of cardiolipin (CL) in the inner membrane. Phosphatic acid (PA) as a precursor reaches the mitochondrial matrix via the PRELID–TRIAP1 complex. The CDP-DAG synthase TAMM41 activates PA with cytidine triphosphate (CTP), producing CDP-DAG. Subsequently, the phosphatidylglycerol phosphate synthase 1 (PGS1) catalyzes the formation of phosphatidylglycerol phosphate (PGP), followed by the dephosphorylation generating phosphatidylglycerol (PG) via the mitochondrial protein-tyrosine phosphatase 1 (PTPMT1). In a last reaction, Cardiolipin synthase 1 (CLS1) mediates the emergence of premature CL based on one molecule of PG and one CDG-DAG. Following the initial synthesis, CL remodeling ensues. After the removement of fatty acids (FAs) mediated by a member of the phospholipase iPLA2 family, the incorporation of new FAs is proceeded by Tafazzin to form matured CL from Monolysocardiolipn (MLCL). To reach an energetic optimum, the remodeling and the ongoing exchange of FAs is a continual persisting process, culminating in a highly diversified CL pool.

Mitochondrial membranes are characterized by their high content of membrane proteins and unique phospholipid composition. A recent study found the respiratory chain, the ADP/ATP carrier, phosphatidylethanolamine, phosphatidylcholine and cardiolipin to be the main constituents of the inner membrane [10]. The hallmark lipid is the dimeric phospholipid cardiolipin (CL), which is found almost exclusively in the inner mitochondrial membrane. Four different fatty acids can be bound to CL forming different CL species, leading to a highly diversified CL pool [11]. CL in the colon has short and mostly saturated fatty acids, whereas CL in the brain primarily constitutes long and unsaturated CL species [12]. The heart has an unique CL pool consisting mostly of tetralinoleoyl-CL (CL(18:2)). Interestingly, the highest proportions of oxidized CL were found in heart (1.8% ± 0.7%) and skeletal muscle [12]. The diversified CL pool is acquired by reshaping CL acyl composition after its initial biosynthesis. CL is synthesized by enzymes located in the inner mitochondrial membrane. An important step in the biosynthesis of CL in the inner membrane is catalyzed by PTPMT1, converting phosphatidylglycerol phosphate to phosphatidylglycerol (Figure 1). Deletion of this in mice is embryonically lethal and PTPMT1-deficient Mouse Embryonal Fibroblasts (MEFs) of embryos obtained from intercrosses of PTPMT1+/flox mice reveal the essential role of this enzyme in CL biosynthesis and affect mitochondrial morphology and respiration [13]. CL Synthase (CLS1) catalyzes the subsequent addition of a second molecule of CDP-DAG to form premature CL (Figure 1). The embryonic lethality of CLS1-deficient animals underscores the essential role of CLS1 in CL biosynthesis. Neuron-specific knockout results in neuronal loss and gliosis in the forebrains as a result of defective respiration and morphological abnormalities in these mitochondria [14].

Afterwards, CL remodeling is initiated by deacylation, catalyzed by mitochondrial members of the Ca^2+^ independent phospholipases to form monolysocardiolipin (MLCL). Subsequently, CL is reacetylated by Tafazzin to form mature CL (Figure 1).

The protein Tafazzin is produced by alternative splicing of the TAZ gene. The gene consists of 11 exons and two ATG initiation sites. Transcription of the TAZ gene gives rise to multiple mRNAs by alternative splicing at exons 5–7. A highly hydrophobic segment of 30 residues at the *N*-terminus acts as a membrane anchor. Tafazzin is directed to mitochondria with the help of two independent targeting sequences [15]. The active site of phospholipid-binding has been identified in a 57-amino acid cleft containing positively charged residues [16]. The substrate specificity of Tafazzin was found to be surprisingly low and cannot explain the tissue-specific remodeling of the CL pool [17]. Recent data suggest that Tafazzin’s substrate specificity is determined by the thermodynamic properties of lipid domains in the vicinity of the enzyme [17]. Tafazzin exchanges fatty acids in the CL molecule until the molecular species composition with optimal packing conditions and the lowest free energy is established [18]. A recent study from the Schlame lab suggests that the thermodynamic properties of lipid domains are determined by the assembly of respiratory chain complexes. This study proposes that protein crowding in the respiratory chain imposes packing stress on the lipid bilayer, which is relieved by CL remodeling to form tightly packed lipid–protein complexes [19]. Further evidence was provided by an integrative approach of lipidomics and transcriptomics. Oemer et al. found that a transcriptional regulation of CL biosynthesis genes shows no correlation with the tissue-specific CL composition. Interestingly, the only significant compliance with CL content was found for genes of the respiratory chain. This finding supports the idea that the respiratory chain and other protein complexes determine the tissue-specific CL composition of the membrane and hence its properties.

Besides Tafazzin, two other enzymes are involved in CL remodeling. The Coenzyme A-dependent monolysocardiolipin acyltransferase (MLCLAT1) is a splice variant of the HADHA gene encoding for the α-subunit of the human trifunctional enzyme (α-TFP) [20,21]. In a recent study, Miklas et al. show that apart from its function in the β-oxidation cycle, α-TFP plays a role in CL remodeling [22]. As patient-derived HADHA knockout cardiomyocytes do not accumulate MLCL as TAZ mutant cells, it has been concluded that HADHA acts subsequently to TAZ to mature the CL pool. The second enzyme is the ER-MAM resident lysocardiolipin acyltransferase (ALCAT1). Overexpression of ALCAT1 in mouse myoblasts increases the incorporation of docosahexaenoic acid (22:6) into CL, yielding a peroxidation prone form of CL, which has been suggested to reflect a pathogenic CL remodeling [23].

## 3. Function of CL in Mitochondrial Morphology

Structural abnormalities of mitochondria, including fragmentation, disruption of mitochondrial membranes and loss of the electron-dense matrix have been observed in animal models of heart failure and in patients suffering from hypertrophic cardiomyopathy [24]. In a dog model of chronic heart failure, the mitochondrial ultrastructure changes from tightly packed to disorganized cristae [25]. Mitochondria possess two highly specialized membranes—the outer and the inner mitochondrial membrane. Invaginations in the inner membrane form the cristae structures, which are areas of intensive membrane curvature. CL plays a considerable role in shaping the morphology of mitochondria and prominently locates in the bended regions of the inner membrane [26]. CL adopts a cone-shaped structure due to the high content of unsaturated fatty acids, which is causative for the membrane bending at sites of high CL concentration. In addition, mitochondrial morphology is also shaped by a constant remodeling through the fission and fusion of individual mitochondria [27]. The opposing processes of fusion (merging of two mitochondria) and fission (segregation of two mitochondria) are shown to form a highly dynamic network of mitochondria in the cell. The continuing remodeling by fission and fusion is essential for normal mitochondrial function, downregulation of mitochondrial fusion promotes apoptosis and cardiomyocyte loss [28]. Mitochondrial fragmentation was elucidated to influence heart failure notably [29]. By its role in regulating mitochondrial fission and fusion, CL actively participates in shaping the mitochondrial network. Fission and fusion are regulated by a set of dynamin-related GTPases, whereby CL serves as a modulator of the activity of the fission protein Drp1 and the fusion protein OPA1 [30,31]. Morphological alterations of mitochondria are evident in human patients with defects in CL biosynthesis and remodeling as well as in animal models (Table 1).

**Figure 2:**
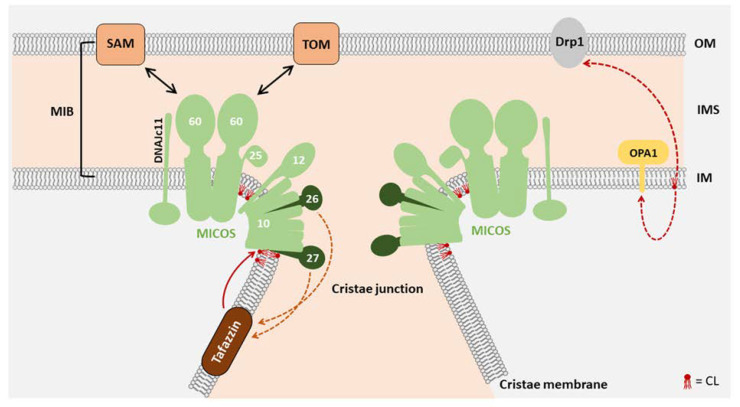
The role of CL in mitochondrial morphology: The mitochondrial contact site and cristae organizing system (MICOS) complex requires CL for optimal structural integrity. MICOS is a protein complex located in the inner mitochondrial membrane, playing an essential role in cristae junction formation. The resulting membrane invaginations harbor the respiratory chain complexes. By interactions with other proteins in the outer mitochondrial membrane such as the Translocase of the Outer membrane (TOM) and the sorting and Assembly Machinery (SAM), the mitochondrial intermembrane space (IMS) bridging complex (MIB) is formed. The MICOS subunits MIC27 and MIC26 influence the regulation of CL levels, while CL is remodeled by Tafazzin [47]. Not only the integration of MIC27 in MICOS, but also the function of other mitochondrial membrane anchored proteins such as the fission and fusion proteins Drp1 and OPA1 is affected by CL [48]. OM, outer membrane; IMS, intermembrane space; IM, inner membrane.

The formation of cristae structures critically depends on the mitochondrial contact site and cristae organizing system (MICOS). These protein complexes locate at the cristae junctions and seal individual cristae to maintain individual membrane potentials [8]. The MICOS complex is a central component of a large interaction network which includes several complexes in the inner membrane and outer membrane [41]. MIC27 (APOOL) as a MICOS complex constituent directly interacts with CL and this interaction was found to be essential for MIC27 assembly in the MICOS complex [42,43] (Figure 2). MIC27 and the structurally related MIC26 also modulate the cardiolipin remodeling enzyme Tafazzin (see below) [44]. In fibroblasts from Barth syndrome patients, a compensatory increase in MICOS subunits was observed [38,45]. Interestingly, a slightly lower molecular mass was detected for MICOS subunits in Barth syndrome (BTHS) fibroblasts, indicative of structural changes due to CL deficiency. Application of the CL-interacting molecule SS31/Elamipretide normalized deregulated structural proteins, such as Drp1, Mfn2, Opa1 and Mic60 in human heart failure patients [46].

## 4. Function of CL in Energy Metabolism

In total, 95% of the cardiac energy demand is covered by oxidative phosphorylation of the respiratory chain in the inner membrane, consisting of five complexes (I–V). Four complexes (I–IV) transport electrons from reducing equivalents (NADH, FADH_2_) to oxygen, forming water. The resulting energy is stored in a membrane potential across the inner membrane and converted to ATP by the last complex in the chain (V). The structure of the respiratory chain has been resolved and specific interaction sites for CL were identified in all respiratory chain complexes [36,49]. Correspondingly, CL is a structural component of the respiratory chain and is essential for its integrity and full enzymatic activity [50,51,52,53]. Changes in the CL pool, including the accumulation of MLCL, may interfere with the structure of respiratory chain complexes. MLCL, which strongly accumulates in Barth syndrome, binds to complex IV with a substantially reduced affinity and significantly lowers the enzymatic activity of this complex [54]. The structure of the dimeric ATP synthase from bovine mitochondria recently shed light upon the mechanism of proton uptake in the matrix. Interestingly, this study also described the incidence of CL in the integral membrane subunits of complex V [55]. Furthermore, CL is directly involved in the proton export and required for the organization of the F_1_F_O_ ATPase into highly ordered structures [56,57].

**Figure 3:**
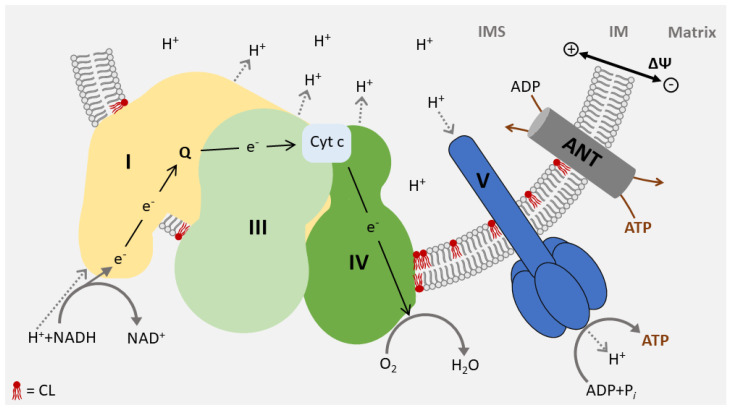
CL is an essential constituent of respirasomes: The mitochondrial respiratory chain assembles into large oligomeric structures called respirasomes. Respirasomes consist of complex I, a dimer of complex III, and several copies of complex IV. CL is required for respirasome formation and is essential for the activity of the complexes. CL molecules partially interacting with membrane protein complexes are shown in red. ANT, ADP/ATP carrier; Cyt c, Cytochrom c; IM inner membrane; IMS, intermembrane space.

The respiratory chain complexes assemble into higher-ordered structures—the respirasomes. These supercomplexes include complex I, a dimer of complex III, and one or several complex IV units (Figure 3). CL molecules are not an only integral to individual complexes but are also associated with the mitochondrial supercomplex formation. It was found to support the structure of supercomplexes and mediate the interaction with the lipid phase of the inner membrane [58,59,60]. Due to its role in the structure of the respirasomes, CL deficiency causes a defect in respiratory function and a decrease in membrane potential and in ATP synthesis [36,37,61] (Table 1).

In many forms of cardiac disease, reduced CL levels, alterations in the CL pool or Reactive Oxygen Species (ROS)-induced damage of CL have been observed. Given the important structural function of CL, these changes may have direct structural implications for the respiratory chain. Remodeling of the respiratory chain due to changes in CL has been described in aging, ischaemia/reperfusion and heart failure [62,63,64,65]. These findings are of particular importance as the architecture of respirasomes prevents ROS production. Structural alterations may induce increased ROS generation at the respirasomes, and increased oxidative stress may be an important contributor to the development of heart failure [66]. Therefore, a large number of studies have focused on preventing mitochondrial ROS in various forms of cardiac disease [67,68]. These studies, although somewhat inconsistent, showed that reducing ROS levels has the potential to ameliorate ROS-mediated cardiac abnormalities [69,70,71].

## 5. Function of CL in Intermediate Metabolism

In order to maintain the membrane potential, the inner membrane must be tightly sealed and transport processes across the membrane need to be vigorously controlled. This contrasts with the demands of energy metabolism, which requires an intensive exchange of metabolites between the cytosol and the matrix. This is ensured by the superfamily of carrier proteins in the inner membrane mediating the transport of metabolites across the inner membrane. The most abundant carrier protein is the ADP/ATP carrier (ANT), which exchanges ATP in the matrix with ADP in the cytosol. The recent resolution of the matrix-open state advances our understanding of the molecular mechanism of metabolite cycling, which most likely applies to the whole carrier family [72]. The ADP/ATP carrier possesses not only a tight binding to CL (Figure 3), but CL was also identified in its crystal structure from bovine heart mitochondria [73,74]. In addition, studies of bakers’ yeast show that the conformation of the ADP/ATP carrier is controlled by CL acting as a link for dimer formation and the integration of ANT into a complex interaction network with the respiratory chain [75]. A requirement for CL has also been documented for other members of the carrier family including the Phosphate carrier (PiC), the monocarboxylate carrier (MCT1), carnitine/acylcarnitine translocase, pyruvate carrier and the tricarboxylate carrier [76,77,78,79,80]. Carrier proteins are integral proteins of the inner membrane, synthesized in the cytosol and then transported across the outer membrane to become subsequently integrated into the inner membrane. Their incorporation depends on the protein Translocase of Inner Membrane TIM22. TIM22 is a protein complex and is integrated in the inner membrane in a CL-dependent manner. Moreover, one structural component of the TIM22 complex is acylglycerol kinase (AGK). Besides its role in protein translocation, it also has a second function as a kinase in the biosynthesis of phosphatidic acid (PA), which serves as a precursor of CL biosynthesis [81,82].

The protein creatine kinase (CK) catalyzes the reversible conversion of creatine and adenosine triphosphate (ATP) into phosphocreatine (PCr) and adenosine diphosphate (ADP) and is expressed in the heart, skeletal muscle, brain and kidney. Phosphocreatine serves as a buffer for rapid regeneration of ATP in tissue with a high energy demand. Mitochondrial creatine kinase (mtCK) is located in the mitochondrial intermembrane space, where it uses the local ATP concentration to generate phosphocreatine. In heart and skeletal muscle, sarcomeric mtCK catalyzes the reverse reaction to regenerate ATP in close proximity of the site of high energy turnover. In-vitro experiments suggest CL as one of the main binding sites of the creatine kinase to the inner membrane [83,84]. mtCK was also found to form large oligomeric complexes in the intermembrane space [85]. By binding to CL, the creatine kinase was also suggested to mediate CL transfer between the inner and outer membrane [86].

Studies in CL-deficient yeast show reduced levels of acetyl-CoA due to a decreased activity of acetyl-CoA synthetase. Despite a compensatory upregulation of pyruvate dehydrogenase (PDH), the enzymatic activity was not increased, suggesting a defect in the specific PDH activity [75]. A C2C12 myoblast model of BTHS confirmed diminished PDH activity and proposed that an increased level of inhibitory phosphorylation was responsible for the defect. The authors suggested that CL is required to facilitate the binding of the pyruvate dehydrogenase phosphatase to the E2 subunit and reduced binding enhances inhibitory PDH phosphorylation [87]. To compensate for the deficiency in PDH activity, the pyruvate carboxylase is upregulated. A defect in PDH activity was not verified in an induced Pluripotent Stem Cell-derived Cardiomyocyte (iPSC-CM) model of BTHS, which even showed an increased flux of glucose into the Krebs cycle intermediate citrate. Accordingly, the anaplerotic supplementation by carboxylation of pyruvate was reduced in this cell model [88].

CL deficiency can also affect the mitochondrial Krebs cycle (TCA). The α-ketoglutarate dehydrogenase complex was found to be structurally affected in human BTHS patient fibroblasts; however, its enzymatic activity and the metabolic flux of glutamate into the Krebs cycle remained unaffected [38]. In addition, a CL-deficient yeast model showed alterations in the enzymatic activities of aconitase and succinate dehydrogenase [87]. Metabolic flux analyses in iPSC-CM displayed an increased level of the Krebs cycle intermediate citrates and decreased level of fumarate in BTHS, indicative of a reduced turnover of succinate into fumarate [88]. Aconitase and succinate dehydrogenase are strictly dependent on iron sulfur clusters as a cofactor. These data and an increase in mitochondrial iron amounts pointed to a defect in the biogenesis of iron sulfur clusters. CL is required for the correct processing of the mitochondrial protein frataxin, which is an important scaffold protein in the iron sulfur cluster biogenesis [89,90]. Consistent with a defect in the TCA cycle, a recent report showed that anaplerotic pathways are required to ameliorate TCA cycle dysfunction in yeast cells [91]. Other cofactors may also be affected. A recent study assumed that lower levels of the cofactor Coenzyme A contribute to the respiratory deficiency in BTHS [92]. Coenzyme A is an important cofactor for fatty acid metabolism. Studies in yeast, however, described that increased fatty acid oxidation can compensate for the Krebs cycle defects in CL-deficient yeast [91]. Under conditions of a high-fat diet, Cole et al. were able to show intensified accumulation of triacylglycerides in cardiac tissue of BTHS mice compared to control animals. The authors found the upregulated synthesis of fatty acid synthase to be responsible for this effect. Increased triacylglycerides may enhance the susceptibility to lipotoxicity in BTHS under conditions of increased fat uptake [93].

Diseases, which are associated with defects in CL, such as DCMA, Sengers syndrome and Barth syndrome, commonly present with 3-methylglutaconic aciduria (3-MGA) accompanied by increased levels of lactic acid [94]. 3-Methylglutaconic and the related 3-methylglutaric acid are catabolic intermediates of the branched-chain amino acid (BCAA) leucine. Additionally, elevated levels of the intermediate of isoleucine metabolism, 2-ethylhydracrylic acid (2-EHA), occurred in the urine of BTHS patients [95]. A few studies allow for speculations of a defect in the metabolism of the branched-chain amino acids valine, leucine and isoleucine in BTHS. Transcriptome analysis of shTAZ mouse model revealed reduced gene expression of genes involved in BCAA breakdown [32]. A recent analysis of protein complexes in the mitochondria of BTHS patient skin fibroblasts revealed a profound destabilization of the branched-chain ketoacid dehydrogenase complex, which is involved in the initial oxidative degradation step of branched-chain amino acids [38]. Further investigations are required to elucidate the molecular mechanism of the observed secretion of organic acids in BTHS.

## 6. CL Function in Calcium Homeostasis

**Figure 4:**
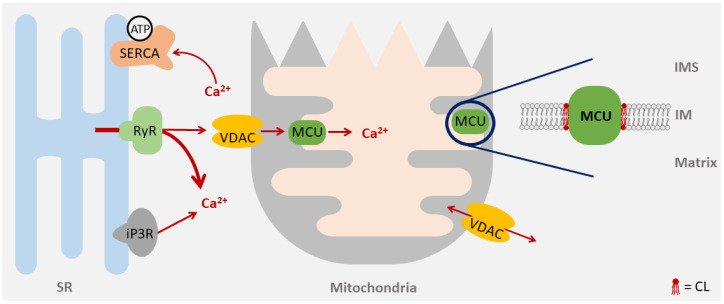
Mitochondrial Calcium Uniporter: Calcium transport from the sarcoplasmic reticulum (SR) is mediated by different proteins—Ryanodine receptors (RyRs) and inositoltriphosphate receptors (iP3Rs) release Ca^2+^ under systolic conditions, while the voltage-dependent anion channel (VDAC) and mitochondrial calcium uniporter (MCU) allow the Ca^2+^ uptake in mitochondria. MCU is embedded in the inner mitochondrial membrane and requires CL for optimal activity. The sarcoplasmic reticulum Ca^2+^-ATPase (SERCA) controls the Ca^2+^ uptake by the SR. IMS, intermembrane space; IM, inner mitochondrial membrane.

Calcium is an important regulator of sarcomere contraction in the heart. In systole, Ca^2+^ influx via L-type Ca^2+^ channels triggers the release of Ca^2+^ from ryanodine receptors (RyRs) in the sarcoplasmic reticulum (SR). Ca^2+^ binding to troponin C induces contraction. During diastole, Ca^2+^ is transported back into the SR by the sarcoplasmic reticulum Ca^2+^-ATPase (SERCA) or exported across the cell membrane via the Na^+^/Ca^2+^ exchanger [96] (Figure 4). By means of the active transport of Ca^2+^ ions, the P-type ATPase SERCA is required for muscle relaxation and the proper regulation of muscle contraction. It also ensures a sufficient Ca^2+^ load in the sarcoplasmic reticulum for systolic contraction. Oxidative stress has been associated with a deregulation of excitation–contraction coupling in the BTHS. Peroxynitrite formed by increased levels of superoxide and nitric oxide (NO) can build adducts with tyrosine residues, which may change the structure or catalytic activity of target proteins [97]. Tyrosine nitrosylation of SERCA in BTHS leads on to a decrease in SERCA activity and results in a decline of SR Ca^2+^ levels. These abnormalities may promote left ventricular diastolic dysfunction. A decrease in SR Ca^2+^ levels has been observed in many forms of heart failure, including dilated cardiomyopathy [98,99].

Calcium is an important regulator of mitochondrial metabolism. Accelerated cardiac workload causes an enhanced demand of ATP. The conversion of ATP to ADP in energy-consuming processes such as contraction increases ADP levels, accelerates respiration and results in an elevated oxidation of reducing equivalents. To compensate for the higher demand for reducing equivalents, Ca^2+^ plays an important role in activating the mitochondrial Krebs cycle [100]. During excitation–contraction coupling, Ca^2+^ is emitted from the sarcoplasmic reticulum and is transmitted into the mitochondria via the Mitochondrial Calcium Uniporter (MCU) (Figure 4). To allow for an efficient calcium transmission, ryanodine receptors (RyRs) in the sarcoplasmic reticulum are located in close proximity to mitochondria, especially next to the mitochondrial calcium uniporter (MCU) in the inner membrane [101]. Ca^2+^ stimulates key dehydrogenases of the Krebs cycle, including pyruvate dehydrogenase, isocitrate dehydrogenase and α-ketoglutarate dehydrogenase, accelerating the regeneration of NADH and FADH_2_. However, the forced regeneration of reducing equivalents activates the respiratory chain. Therefore, Ca^2+^ plays a prominent role in adapting mitochondrial metabolism to increased energy demands during accelerated cardiac workload [102]. The pore-forming unit of the mitochondrial calcium uniporter is the protein MCU, which associates EMRE and regulatory subunits MICU1, MICU2, and MCUb into a complex integrated into the inner membrane [103,104,105,106] (Figure 4). The association of phospholipids in MCU has been revealed in a recent structural analysis [107]. Moreover, a specific requirement of the MCU for CL was found. Consequently, the ability of MCU to assemble into functional complexes was reduced in cardiac patient samples of BTHS [108].

## 7. Barth Syndrome

**Figure 5:**
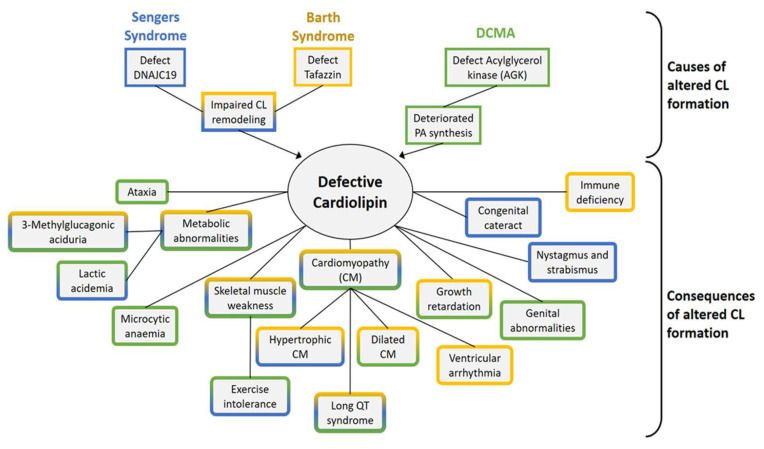
Clinical alterations, caused by a defect in the CL pool: Colors indicate clinical symptoms matching Sengers syndrome (blue), Barth syndrome (yellow) and dilated cardiomyopathy with ataxia (DCMA, green).

BTHS patients have a quite variable clinical presentation of cardiomyopathy ranging from milder cases to severe cases, which require cardiac transplantation. BTHS patients present with a dilated or hypertrophic cardiomyopathy and left ventricular non-compaction [109,110,111,112]. The left ventricular ejection fraction (LVEF) was only moderately reduced—50 ± 10% (normal LVEF 50–70%, mild dysfunction LVEF 40–49%, moderate dysfunction LVEF 30–39% and severe dysfunction LVEF <30%) [111,112]. Despite preserved LVEF, many patients show an inability to increase cardiac output during exercise [113]. A very common symptom is skeletal muscle fatigue and exercise intolerance [110,113]. BTHS patients also manifest metabolic abnormalities in the degradation pathway of branched amino acids and show lactic acidosis during exercise as well as an elevated excretion of 3-Methylglutaconic acid (3-MGA) in urine [113,114] (Figure 5). Defects in the immune system include persistent or intermittent neutropenia causing recurrent infections. A growth deficiency during childhood is compensated by a delayed growth spurt after 12–14 years of age in BTHS [111,112,115].

BTHS is frequently associated with a severe exercise intolerance, which makes it difficult to perform activities of daily living and drastically reduces quality of life. Skeletal muscle impairments include harmed functional exercise capacity, diminished extensor strength, and lowered daily activity [116]. Exercise intolerance is thought to be a consequence of cardiac impairment and decreased skeletal muscle oxygen utilization [113]. Changes in CL levels also in the skeletal muscle of Barth syndrome patients cause the destabilization of respirasomes, reduction in respiration, excessive production of reactive oxygen, abnormal mitochondrial morphology and defects in ATP production [115,117,118]. The development of a cardiac-specific knockout model recapitulated cardiomyopathy with reduced fractional shortening, increased hypertrophy and left ventricular dilatation. A constitutive knockout of Taz, however, resulted in very poor survival. Interestingly, the low survival rates were rescued, upon skeletal muscle-specific virus transmitted gene replacement therapy, indicating a particular contribution of skeletal myopathy to the reduced survival of the mice [39].

Using ^31^P nuclear magnetic resonance (NMR) spectroscopy, a higher content of glycolytic fibers (type 2, fast-twitch) and a smaller fraction of oxidative fibers (type 1, slow-twitch) has been documented in the skeletal muscle of BTHS patients [119,120]. This finding suggests that BTHS patients rely on glycolytic metabolism to a greater extent than control individuals. Moreover, a higher respiratory exchange ratio during exercise and a greater glucose rate of disposal during a hyperinsulinemic–euglycemic clamp procedure indicate higher glucose usage to compensate for the impaired mitochondrial capacity to generate ATP [113,121]. Interestingly, exercise intolerance has also been described in patients with chronic acquired heart failure [122].

BTHS is caused by mutations in the X chromosomal gene encoding for Tafazzin (TAZ) [123]. Pathogenic mutations in TAZ include frameshift, splice-site, missense, and non-sense mutations [124]. Mutations are associated with a decrease in the amount of functional enzymes, mislocalization of the protein in the cell, protein aggregation or altered macromolecular complex assembly [124,125]. Protein levels and messenger RNA of cardiolipin synthase (CLS), Tafazzin (TAZ) and acyl-CoA:lysocardiolipin acyltransferase-1 (ALCAT-1) are affected in cardiac tissue of BTHS patients. Loss of Tafazzin function causes a significant alteration in the CL pool including a reduction in mature forms of CL and an increase in MLCL, which also serves as a diagnostic marker for BTHS [126,127]. Decreased respiration and reduced activity of single respiratory enzymes have been confirmed in several models of BTHS, including BTHS patient-derived fibroblasts and lymphoblasts as well as cellular and animal models [36,40,128].

BTHS patients have a significantly lower body weight and fat free mass [121]. When measured under resting conditions, the fatty acid oxidation rate related to body mass in BTHS patients was comparable to the control group. However, under exercise conditions the elevated fatty acid oxidation rate was severely blunted in BTHS [121]. In turn, glucose turnover was already increased at rest. Therefore, the metabolic derangements, in particular the inability to upregulate fatty acid metabolism, reflect the exercise inability of BTHS patients [129]. Abnormal blood levels of amino acids were determined in BTHS patient [94,130]—in particular, amino acids, which are associated with anaplerosis in the intermediate metabolism (arginine, ornithine and citrulline), were consistently lower. This strongly supports the hypothesis of alterations in the energy metabolism in BTHS. Increased levels of tyrosine, proline, and asparagine compared to the control were found under starvation conditions [94,130]. Considering that the lower lean and skeletal muscle mass of BTHS patients, the unchanged rates of appearance of the ketogenic amino acid leucine upon starvation is remarkable and led to the hypothesis of a higher proteolysis rate in BTHS patients in order to use amino acids for energy needs [121].

## 8. Sengers Syndrome

The clinical manifestation of Sengers syndrome is hypertrophic cardiomyopathy and congenital cataracts. Other symptoms including skeletal myopathy, exercise intolerance, lactic acidosis and 3-methylglutaconic aciduria, showing strong similarities to Barth syndrome [131] (Figure 5). Sengers syndrome is caused by mutations in the gene encoding for mitochondrial acylglycerol kinase (AGK) [132]. AGK is a mitochondrial lipid kinase strongly expressed in heart, but also in the skeletal muscle, kidney and brain. This protein can phosphorylate monoacylglycerol to lysophosphatidic acid (LPA) and diacylglycerol to phosphatidic acid (PA), a precursor of CL biosynthesis [132]. AGK has an additional independent function in mitochondrial protein import as a constituent of the mitochondrial carrier translocase TIM22 complex [81,133]. This inner membrane-embedded protein translocase imports proteins of the carrier family from the cytosol. Carrier proteins are a large family of proteins which mediate the transport of metabolites across the inner membrane. A defect in the import and assembly of carrier proteins has been found in AGK-deficient mitochondria [81,133]. In particular, decreased levels of mitochondrial ADP/ATP carrier in heart and muscle tissues was the initial finding in patients of Sengers syndrome [134]. The function in protein transport is independent of AGK kinase activity. It can be assumed that loss of both functions contributes to the pathogenesis in Sengers syndrome.

## 9. Dilated Cardiomyopathy with Ataxia (DCMA)

Mutations in the gene encoding for the mitochondrial protein DNAJC19 were found to be causative for Dilated Cardiomyopathy with Ataxia (DCMA) [135]. DCMA patients presenting with dilated cardiomyopathy and arrhythmias due to abnormalities in repolarization after a heartbeat (long QT syndrome, LQTS) [136]. Similar to BTHS and Sengers syndrome, 3-methylglutaconic aciduria is commonly described in DCMA (Figure 5). Other symptoms include cerebellar ataxia, growth retardation or genital anomalies in male patients [137]. Patient-derived iPSC-CM models of DCMA have been developed recently [138,139]. The function of the affected gene (DNAJC19) is unknown but it shares sequence similarities with the family of DnaJ proteins, which act as cofactors of Hsp70 chaperones. DNAJC19 binds to the mitochondrial protein prohibitin which is integrated in the inner membrane and oligomerizes into large ring-like structures, which restrict CL into specific membrane domains. The role of prohibitin appears to be to segregate specific membrane domains, which facilitate CL remodeling. The direct interaction of DNAJC19 with prohibitin suggests a participation of DNAJC19 in CL remodeling. In fact, deletion of DNAJC19 in a cell model resulted in changes in the CL species composition [140]. Interestingly, these alterations in the CL pool were not approved in an iPSC-CM model of DCMA [139]. The reason for these inconsistencies have not been resolved yet. A consistent finding, however, is that DNAJC19 deficiency causes highly fragmented and abnormally shaped mitochondria and changes in mitochondrial cristae morphology.

## 10. Therapeutic Approaches

Improvements in the therapy of BTHS cardiomyopathy and the associated symptoms such as neutropenia and skeletal myopathy have resulted in improved survival of the deadly disease. Standard therapy addressing the cardiac defects are angiotensin-converting enzyme (ACE) inhibitors or angiotensin receptor blockers, in combination with beta-adrenergic receptor blockers [141]. More severe cases are treated with vasodilators or inotropes, left ventricular assist devices, and/or cardiac transplantation [142,143,144]. Neutropenia is treated with granulocyte colony-stimulating factor (G-CSF) and complemented with prophylactic antibiotics. Growth hormone (GH) supplementation is used to treat the growth delay and arginine supplementation acts as a complement for arginine depletion [141,145,146].

Gene therapy in inherited diseases allows to directly target affected genes and has been tested in mouse models of Barth syndrome. Adeno-associated virus (AAV)-mediated *TAZ* gene replacement ameliorated cardiac function in *Taz*-KD mice, indicating the reversibility of the clinical phenotype and the feasibility of the approach [147]. Additionally, pharmaceutical intervention in CL biosynthesis has been tested in experimental studies. The mitochondrial enzyme phospholipase A_2_ is responsible for the deacylation of premature CL after its initial synthesis. The resulting monolysocardiolipin MLCL is then acetylated to mature CL by Tafazzin [148]. Inhibiting phospholipase A_2_ would result in stabilizing the premature CL pool and prevents the accumulation of MLCL. This intervention has been tested in a *Taz* deletion Drosophila model of BTHS. *Taz* deletion causes male sterility in flies, which was prevented by inhibiting phospholipase A_2_ [148]. However, as the human homolog of the *Drosophila* phospholipase A_2_ has not been identified and its inhibition might have severe side effects, translation into medicine is difficult [149].

Therapeutic approaches are interesting with regard to the targeting of metabolic defects in BTHS. Due to a central role in energy metabolism and mitochondrial bioenergetics, peroxisome proliferator-activated receptors (PPARs) have been considered as potential therapeutic targets. Activation of the PPAR/PGC1α axis using the PPARα agonist bezafibrate has been a successful strategy in various mitochondrial disorders [150]. PPARα activation regulates the transcription of numerous genes involved in mitochondrial energy metabolism and fatty acid oxidation [151,152]. When tested in the BTHS mouse model, bezafibrate prevented the development of systolic dysfunction and improved exercise capacity when combined with voluntary exercise [153].

As the structural change in the respiratory chain in Barth syndrome is associated with a marked increase in ROS emission from mitochondria [36,154,155], an intriguing therapeutic strategy is the use of anitoxidants. The mitochondria-targeted ROS scavenger mitoTEMPO was tested in the human-induced pluripotent stem cell (iPSC)-derived cardiomyocytes of BTHS patients [156,157]. ROS-induced changes in sarcomere assembly and the resulting deficits in contractility undergo an improvement with mitoTEMPO [158]. The translation into a mouse model was tested by expressing the ROS-scavenging catalase in the mitochondrial matrix to target mitochondrial hydrogen peroxide (H_2_O_2_) emission in the BTHS mouse. This approach efficiently reduced H_2_O_2_ levels and lipid peroxidation, but did not eradicate cardiac dysfunction or skeletal muscle fatigue in *Taz*-knockdown (KD) mice [159]. The discrepancy of these contradictory results has not been resolved, yet.

Cytochrome c mediates electron transfer from complex III to complex IV in the respiratory chain and is also involved in the peroxidation of cardiolipin (CL), which has been observed in a variety of pathological conditions, including BTHS [160]. Peroxidized CL is associated with energy deficiency and plays a role in the opening of the permeability transition pore (PTP) [161]. The PTP is a large pore in the mitochondrial membranes and consists of the proapoptotic Bcl-2 members Bax/Bak in the outer membrane and the F_1_/F_O_ ATPase in the inner membrane and cyclophilin D in the matrix [162]. PTP opening causes a depletion of the membrane potential and induces apoptosis. The Szeto-Schiller peptide (SS-31 or Elamipretide) is an aromatic-cationic mitochondria-targeting tetrapeptide that penetrates the plasma membrane and localizes to the inner mitochondrial membrane based on its direct interaction with CL. Elamipretide prevents the peroxidase activity of cytochrome c and normalizes the CL pool in models of BTHS and other models of heart failure [163,164]. Elamipretide also improves inner membrane cristae structures and re-establishes mitochondrial respiration and ATP production in models of heart failure including BTHS [165,166]. Unfortunately, a phase II clinical trial (TAZPOWER trial, NCT03098797) treatment with Elamipretide in BTHS patients did not improve exercise capacity. Recently, Elamipretide was also tested in fibroblasts from DCMA patients and was found to rescue mitochondrial fragmentation and increased ROS production [167]. Heart tissue from a rat model of ischemia-reperfusion showed deterioration of mitochondrial complexes I, II, and IV. Application of Elamipretide significantly alleviated the structural changes in respirasomes, improved the fragmentation of mitochondria and enhanced the formation of cristae structures [168]. As changes in the CL pool were also found in the aging heart, Elamipretide was tested in aging mice. Here, it reduced mitochondrial ROS and normalized protein oxidation in old hearts, and even showed beneficial effects for aging-related diastolic defects. Interestingly, expression of the mitochondrial catalase presented similar beneficial effects, which were not further improved by Elamipretide application, indicating normalizing mitochondrial oxidative stress as the main mechanism for Elamipretide in aging hearts [67].

## 11. Conclusions

Mitochondria play a crucial role in energy metabolism, redox homeostasis and intermediate metabolism, not only having additional anabolic functions but also participating in signaling pathways. Many of these functions are membrane-associated and were shown to be dependent on CL. CL-deficient cells and animal models, including BTHS patient-derived lymphoblasts, *Drosophila*, *C.elegans*, *Trypanosoma* and mice, have been developed [35,169,170,171,172,173]. These model systems have helped to understand the role of CL in mitochondrial biogenesis and in shaping mitochondrial morphology. CL is an integral component of the respiratory chain since CL deficiency causes a decline in respiratory capacity and an increase in ROS production. A defect in MCU uncouples mitochondrial energy metabolism from myocardial Ca^2+^ signaling, which mediates an important response during accelerated workload conditions. Deficiencies in the Krebs cycle require remodeling of intermediate metabolism for compensatory anaplerotic pathways. Finally, defects in the biogenesis of iron sulfur clusters and Coenzyme A might endanger fatty acid oxidation, one of the most prevalent energy sources for the heart. The tight interaction between CL and membrane proteins, such as respirasomes, may also have implications on the half-life of CL itself, although CL presents a longer half-life compared to other phospholipids [118,174,175]. As the slow turnover of CL is dependent on its interaction with the respiratory chain, respiratory chain remodeling as in BTHS may induce a vicious cycle when increased CL turnover may contribute to low CL levels in Barth syndrome [118]. The exact mechanisms of CL degradation remain unclear. The phospholipase HSD10 has been suggested to mediate rapid degradation, particularly of oxidized CL (CL^OX^) [176]. Patients with mutations in the gene encoding HSD10 suffer from progressive cardiomyopathy and neurodegenerative disease. The contribution of HSD10-mediated CL^OX^ clearance for the maintenance of functional mitochondria has not been studied yet.

Barth syndrome patients suffer from a general metabolic remodeling including changes in serum amino acid levels, lactic acidosis during exercise and elevated urinary excretion of 3-MGA [113,114]. In the future it will be interesting to understand the exact mechanisms behind changes in the metabolism, how they relate to mitochondrial dysfunction and how dysfunctional mitochondria induce compensatory mechanisms such as anaplerotic pathways. Based on the integration of mitochondria in cellular signaling pathways, changes in mitochondrial function might be monitored and trigger a cellular response. ROS may be at the center of this mechanism since alteration in ROS according to CL deficiency has been widely documented. Being a part of a large number of cellular signaling pathways, ROS affect a wide variety of biological processes including responses to hypoxia, apoptosis, autophagy, cell proliferation and differentiation. Alterations in ROS signaling due to CL deficiency cause a defect in key cellular signaling pathways involved in the response to hypoxia [155]. Several signaling pathways are also directly dependent on CL. Kinases of the protein kinase C (PKC) family regulate diverse biological functions such as growth and differentiation and influence multiple physiological processes in the heart, including heart rate, contraction, and relaxation. Members of the PKC family locating to the mitochondria and requiring CL for activation have been described previously [177,178]. As CL predominantly is located in the inner membrane, externalization of CL onto the outer membrane serves as a signaling platform in many signaling events, such as mitophagy and apoptosis. How these changes relate to the clinical picture in heart disease is not understood and remain a challenging task for the future.

## Figures and Tables

**Table 1:** Role of CL in inherited cardiomyopathies in animal models and human.

Function	Anmial	Human
Mitochondrial morphology	A tafazzin knockdown mouse model of Barth syndrome describes alteration of mitochondrial phospholipid compositions via lipidomics [32]. Morphology alterations as mitochondrial enlargement, concentric layers of cristae or large vacuoles were observed in tafazzin-deficient mice [33].	BTHS patient-derived lymphoblasts (BTHS lymphoblasts) reveal enlarged mitochondria with a lower surface area of cristae and altered morphology [34]. Another study with BTHS lymphoblasts detected giant, partly onion-shaped mitochondria [35].
Oxidative phosphorylation	A BTHS mouse model with an inducible systemic knockdown of tafazzin gene shows a reduced respiration on succinate as well as on pyruvate and malate. Furthermore, respirasome remodeling was detected [36].	BTHS patient-derived induced pluripotent stem cells (BTHS-iPSC) reveal structural remodeling of respiratory chain complexes resulting in decreased mitochondrial respiration [37]. In a second study, mitochondria of BTHS lymphoblasts show a reduced respiratory activity on succinate and ascorbate [34].
Krebs cycle	BTHS mouse model reveals a striking reduction in succinate dehydrogenase activity in cardiac mitochondria [36].	BTHS skin fibroblasts reveal a significant destabilization of 2-oxoglutarate dehydrogenase and branched-chain ketoacid dehydrogenase [38].
Apoptosis	Murine germline TAZ knockout mice model reveals significant increased cardiomyocyte apoptosis and fibrosis occurrence [39].	BTHS lymphoblasts show a requirement of cardiolipin for apoptosis and an apoptotic defect [40].
